# Activated platelet membrane nanovesicles recruit neutrophils to exert the antitumor efficiency

**DOI:** 10.3389/fchem.2022.955995

**Published:** 2022-08-11

**Authors:** Yinghui Shang, Juntao Sun, Xin Wu, Qinghai Wang

**Affiliations:** ^1^ Department of Hematology, Shanghai General Hospital, Shanghai Jiao Tong University School of Medicine, Shanghai, China; ^2^ Department of Gastroenterology, The Second Hospital, Cheeloo College of Medicine, Shandong University, Jinan, China; ^3^ Department of Spine Surgery, The Third Xiangya Hospital, Central South University, Changsha, China; ^4^ Department of Cardiology, The Second Hospital, Cheeloo College of Medicine, Shandong University, Jinan, China

**Keywords:** platelet membrane, nanovesicle, neutrophil, antitumor, immunity

## Abstract

Platelets play a crucial role in the recruitment of neutrophils, mediated by P-selectin, CCL5, and ICAM-2. In this study, we prepared platelet membrane nanovesicles from activated platelets. Whether activated platelet membrane nanovesicles can recruit neutrophils has not been reported, nor has their role in antitumor immunity. The results of SDS-PAGE showed that the platelet membrane nanovesicles retained almost all the proteins of platelets. Western blotting showed that both the activated platelets and the platelet membrane nanovesicles expressed P-selectin, ICAM-2, and CCL5. *In vivo* results of a mouse model of breast cancer-transplanted tumor showed that tumor volume reduced significantly, Ki-67-positive tumor cells decreased, and TUNEL-positive tumor cells increased in tumors after treatment with activated platelet membrane nanovesicles (aPNs). After treatment with aPNs, not only the number of neutrophils, CD8^+^, CD4^+^ T cells, and B cells increased, but also IL-12, TNF-α, and IFN-γ levels elevated significantly in tumor tissues.

## 1 Introduction

Neutrophils exhibit significant antitumor effect and can prime anticancer immunity (Ustyanovska Avtenyuk et al., 2020). Neutrophils control the vital part of the adaptive immune system, regulating the function of B and T cells (Liew and Kubes, 2019), stimulating T-cell-mediated antitumor response (Eruslanov et al., 2014). One study showed that CD8^+^, CD4^+^ T cells, and B cells are positively correlated with the reduction of tumor volume (Cunha et al., 2012). There is evidence that neutrophils can cross-present neoantigens to T lymphocytes via major histocompatibility complex (MHC), leading to the initiation of antitumor T-cell response (Singhal et al., 2016), and stimulating T-cell proliferation and activation (Eruslanov et al., 2014). In addition, neutrophils recruit and activate T cells by secreting cytokines, such as TNF-α, cathepsin G, and neutrophils elastase (Mishalian et al., 2014).

Platelets perform a vital part in recruiting neutrophils through self-expressed P-selectin (Nagao et al., 2007). In addition, they also express intercellular adhesion molecule-2 (ICAM-2) and CCL5, and recruit neutrophils mediated by P-selectin glycoprotein ligand 1 (PSGL-1), CCL receptor 1/CCL receptor 5 (CCR1/CCR5), and lymphocyte function-associated antigen 1 (LFA1) (Weber and Springer, 1997; Kuijper et al., 1998; von Hundelshausen et al., 2007).

Although biodegradable and clearable inorganic nanomaterials show progress in cancer theranostics (Wang et al., 2021), bionics and targeted nanotechnology still play an irreplaceable role. It has been reported that activated platelet membrane nanovesicles camouflaging black phosphorus quantum dots can target tumor tissue (Shang et al., 2019). Whether activated platelet membrane nanovesicles alone can target tumor sites and whether targeting tumor sites is beneficial for recruiting neutrophils to the tumor site has not been reported. In this study, we applied the tumor-transplanted mouse model and intravenously injected activated platelet membrane nanovesicles to investigate the mechanism of enhancing antitumor immunity by recruiting neutrophils, thus exploring a new perspective for tumor treatment.

## 2 Materials and methods

### 2.1 Materials

Thermo Fisher Technology Co., Ltd. provided the fetal bovine serum (FBS). Solarbio Biotechnology Co., Ltd. provided RPMI-1640, phosphate buffer saline (PBS), trypsin, Cy5, and non-pre-stained protein marker. Bovine serum albumin (BSA) was procured from Yeasen Biological Technology Co., Ltd. BCA Protein Quantitation Kit and Coomassie Blue Fast Staining Solution were from Beyotime Biology Co., Ltd. Polyvinylidene fluoride (PVDF) membrane was produced by Cell Signaling Technology, Co., Ltd. P-selectin rabbit primary antibody was provided by Shanghai Lianshuo Biological Technology Co., Ltd. ICAM-2 (F-5) mouse primary antibody was purchased from Santa Cruz Biotechnology Co., Ltd. CCL5 rabbit primary antibody was provided by Solarbio Biotechnology Co., Ltd. β-Actin (60008-1-Ig) mouse primary antibody was provided by Proteintech Group, Inc. Servicebio Biological Technology Co., Ltd. provided hematoxylin and eosin (HE), Ki-67 immunofluorescence detection kit, and terminal deoxynucleotidyl transferase-mediated dUTP nick-end labeling (TUNEL) immunofluorescence detection kit. Mouse IFN-γ ELISA kit (E-EL-M0048c) and IL-12 ELISA Kit (E-EL-M0726c) were provided by Elabscience Biological Technology Co., Ltd. Mouse TNF-α ELISA kit (ab208348) was purchased from Abcam Trading Co., Ltd. Ly6G (1900–02) mouse primary antibody was provided by SouthernBiotech Co., Ltd. CD4 (CL647-65104) and CD8 (CL488-65069) fluorescent antibodies were produced by Proteintech Group, Inc. CD19 (ab245235) mouse primary antibody and CD11b (ab24874) mouse primary antibody were produced by Abcam Trading Co., Ltd.

### 2.2 Cell line and mice

4T1 cells were provided by the Cancer Institute of Central South University and cultured in RPMI-I640 medium containing 10% FBS under the condition of 37°C and 5% CO_2_. The Hunan Slake Jingda Laboratory Animal Co., Ltd. provided the female ICR mice of 6-week-old.

### 2.3 Isolation of PLTs and preparation of PLTm nanovesicles

A sample of whole blood from female ICR mice was collected in tubes containing heparin anticoagulant. The whole blood was centrifuged and washed to obtain platelets. The platelets were repeatedly frozen and thawed to obtain PLTm, and then PLTm nanovesicles were generated by ultrasonic treatment (2 min, 42 kHz, 100 W).

### 2.4 Identification of proteins of PLTs and PLTm nanovesicles

SDS-PAGE was applied to display the protein expression profile of PLTs and PLTm nanovesicles. Western blotting was used for the detection of the expression of specific proteins (P-selectin, ICAM-2, and CCL5) on PLTs and PLTm nanovesicles. RIPA lysis buffer containing protease inhibitor cocktail was used to lyse PLTs and PLTm nanovesicles. After that, the lysates were centrifuged at 4°C (13000g, 5 min) and the total protein in the supernatant was measured using the BCA protein detection kit. SDS loading buffer was added to the supernatant, and the total protein was degenerated by heating them at 100°C for 5 min. A quantity of 40 μg total protein was loaded into each gelatin well, and electrophoresis was performed. The gelatin was quickly dyed with Coomassie blue staining solution and photographed. The proteins were transferred to polyvinylidene fluoride (PVDF) membranes and sealed with 5% skimmed milk in TBST at room temperature for 1 h. After that, these blots were incubated with P-selectin, ICAM-2, CCL5, and β-actin primary antibody for 2 h; washed with TBST; incubated with corresponding horseradish peroxidase-linked secondary antibody; washed with TBST; and added with chemiluminescent solution for development with Gel imaging system (ChemiDoc MP, Bio-Rad Laboratories, United States).

### 2.5 Evaluation of *in vivo* targeting of PLTm nanovesicles

Female ICR mice were subcutaneously inoculated with 4T1 cells (1.0×10^6^/100 μl). When the tumor volume exceeded 100mm^3^, dilute solution of Cy5 in PBS and Cy5 (red fluorescence)-labeled PLTm nanovesicles were intravenously injected, respectively. After 48 h, we gleaned the vital organs, including the hearts, livers, spleens, lungs, kidneys, brains, and tumors of the mice, and evaluated the fluorescence intensity by animal imaging (IVIS Spectrum, PerkinElmer, United States). Tumor tissue sections were photographed by using a confocal laser scanning microscope (CLSM) (TCS SP8, Leica, Germany).

### 2.6 Evaluation of the antitumor activity of PLTm nanovesicles *in vivo*

Female ICR mouse were subcutaneously inoculated with 4T1 cells (1.0×10^6^/100 μl). When the tumor volume exceeded 100 mm^3^, PBS and aPNs were injected into the tail vein, respectively, once a day for four times. The length and width of the tumors were measured every 4 days to calculate tumor volume and assess antitumor activity. Tumor volume was calculated as: length × width^2^/2. At day 20, the mice were killed, and the collected tumor samples were stored in 4% paraformaldehyde and then made into tissue sections.

### 2.7 Ki-67 and TUNEL immunofluorescence assays in tumor tissues

Paraffin sections of the tumor tissue were dewaxed, and antigen repair was performed. The tissue sections were covered with membrane-breaking working solution for 20 min and washed with PBS.

#### 2.7.1 Ki-67 immunofluorescence assay

An appropriate sealing solution was added to the slides and incubated in a wet box for 60 min. Anti-Ki-67 rabbit antibody was added to each sample and incubated overnight at 4°C. Then, the slides were removed from the refrigerator at 4°C and washed with PBS for three times, 5 min each time. A quantity of 50 μl of Cy3-conjugated Goat anti-Rabbit IgG was added to each slide, which was incubated away from light for 60 min, washed with PBS, and then, redyed with DAPI.

#### 2.7.2 TUNEL immunofluorescence detection

Reagent 1 (TdT) and Reagent 2 (dUTP) were added, and the sections were incubated at 37°C for 2 h and washed with PBS. Following this, the PBS was removed and DAPI was added, and the slides were incubated for 10 min to avoid light. The slides were sealed with anti-fluorescence quencher and observed, and its images were collected by using a confocal laser scanning microscope (CLSM) (TCS SP8, Leica, Germany).

### 2.8 Immunofluorescence detection of immune cells in tumor tissues

After fixation, embedding, and sectioning, tumor tissue sections were stained with CD8^+^ T lymphocytes (CD8), neutrophils (CD11b, Ly6G), B lymphocytes (CD19), and CD4^+^ T lymphocytes (CD4) primary immunofluorescence antibody for 1 h in dark, washed with PBST, incubated with secondary antibody, washed with PBST for 3 times, and re-dyed with DAPI. The cells were observed, and images were obtained under CLSM.

### 2.9 The level of cytokines in tumor tissues detected with ELISA kits

A standard and sample diluent was added to the tissue samples according to the instruction. The wells were sealed and incubated at 37°C for 90 min, and the plate was washed. Biotinylated antibody diluent or biotinylated antibody working solution was added, the wells were sealed and incubated at 37°C for 60 min and then washed. An enzyme-conjugated diluent or enzyme-conjugated working solution was added, and the wells were sealed and incubated at 37°C for 30 min, and washed. A color substrate was added and the wells were incubated at 37°C for 15 min. A reaction-stopping solution was added, and OD450 was measured immediately. The best fitting curve was drawn, and the concentration of the samples was calculated.

## 3 Results and discussion

### 3.1 Characteristics of PLTm nanovesicles

**FIGURE 1:**
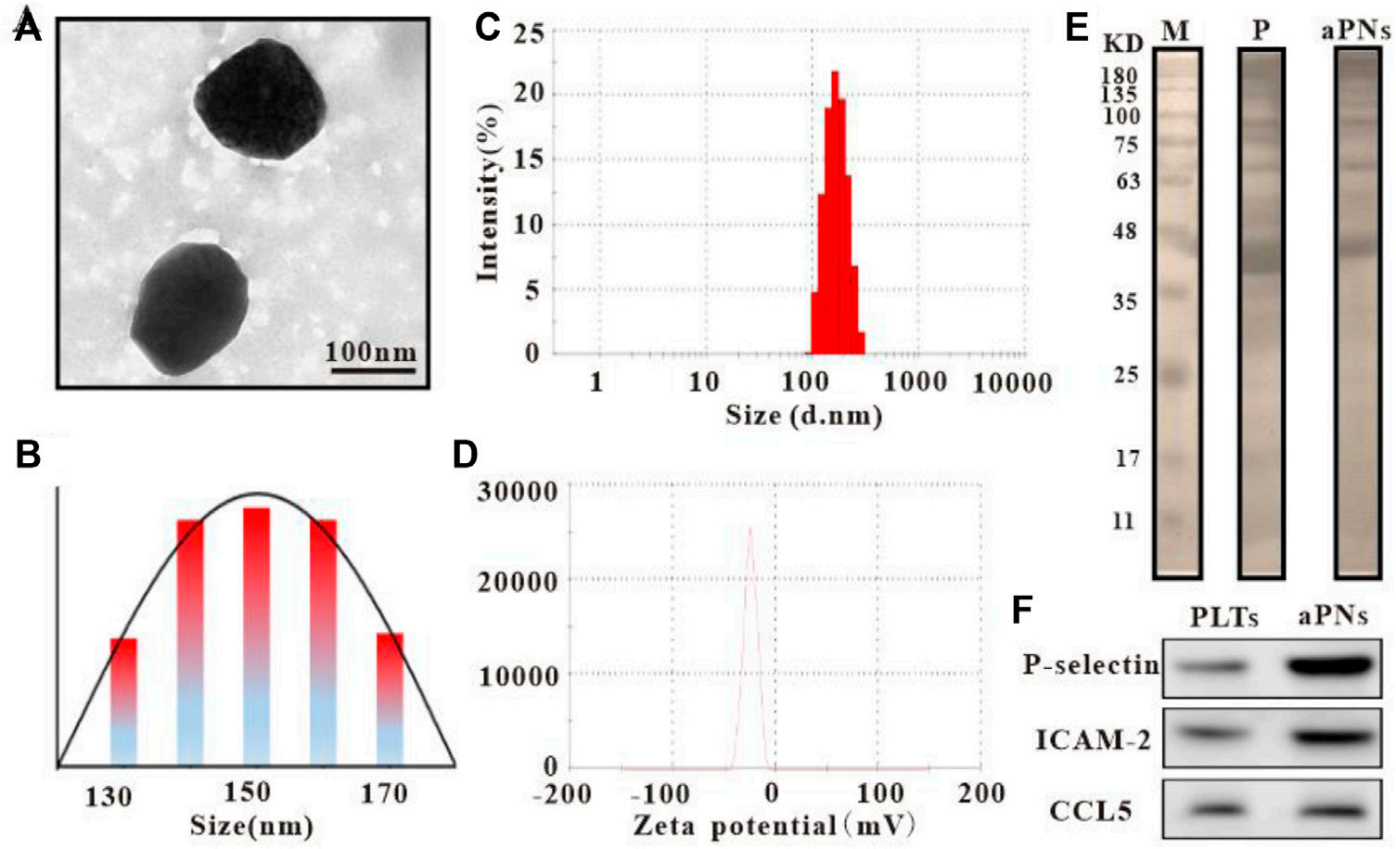
Characteristics of PLTm nanovesicles. **(A)** PLTm nanovesicles observed under TEM, scale bar: 100 nm. **(B)** TEM size distribution of PLTm nanovesicles. **(C)** DLS of PLTm nanovesicles. **(D)** Zeta potential of PLTm nanovesicles. **(E)** SDS-PAGE protein analysis of PLTs and activated PLTm nanovesicles. M: Marker, P: PLTs, aPNs: activated PLTm nanovesicles. **(F)** The expression of proteins (P-selectin, ICAM-2 and CCL5) in PLTs and activated PLTm nanovesicles were analyzed by Western blot. aPNs: activated PLTm nanovesicles.

Observed under transmission electron microscope (TEM) (Tecnai G2 Spirit, Thermo Fisher, United States), the diameter of activated PLTm nanovesicles was about 150 nm (Figures 1A,B), which was similar to the result of dynamic light scattering (DLS) (Figure 1C). Data from Zetasizer Nano ZS (Malvern Nano series, Malvern, United Kingdom) showed that Zeta potential of activated PLTm nanovesicles was about –21.7 mV (Figure 1D). SDS-PAGE protein analysis showed that PLTs expressed more proteins than aPNs, and aPNs retained most of the PLTs’ proteins (Figure 1E). Western blotting showed that P-selectin, ICAM-2, and CCL5 were expressed both in PLTs and aPNs (Figure 1F). Expression of these proteins provided the basis for targeting tumor tissues and recruiting neutrophils.

### 3.2 *In vivo* targeting of activated PLTm nanovesicles

**FIGURE 2:**
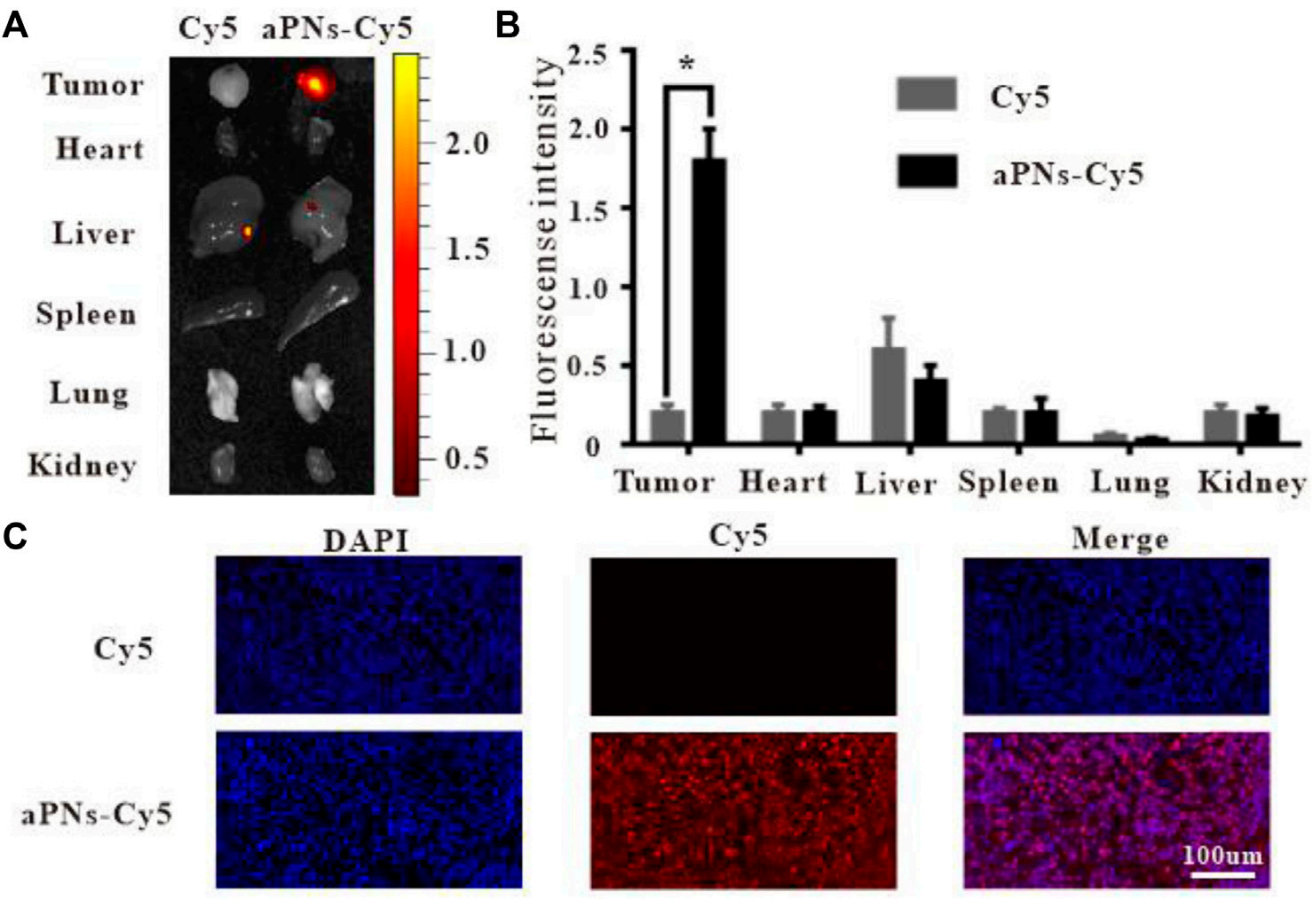
*In vivo* targeting of aPNs. **(A)** Fluorescence images of internal organs and tumor tissues after treatment with Cy5 and aPNs-Cy5. **(B)** Semi-quantitative analysis of organ fluorescent signal, compared with Cy5 group: ^∗^ *p* < 0.05. **(C)** Fluorescent images of tumors from ICR mice at 48 h after injection of Cy5-conjugated aPNs and Cy5, scale bar: 100 μm.

To verify the tumor-targeting properties of activated PLTm nanovesicles, we labeled activated PLTm nanovesicles with Cy5 and assessed their distribution *in vivo*. PBS-diluted Cy5 solution served as control. At 48 h after intravenous injection, retention of aPNs-Cy5 (red fluorescence) in tumor was more obvious than that of Cy5 alone. Moreover, there was no significant accumulation in the two groups in visceral tissues, including heart, spleen, lung, and kidney, except for a small amount of fluorescence in the liver (Figures 2A,B). In addition, the bright red fluorescence in tumor tissues after injection of aPNs-Cy5 was more obvious than that after the injection of Cy5 alone (Figure 2C), indicating that aPNs possess good tumor-targeting property.

### 3.3 *In vivo* antitumor activity

**FIGURE 3:**
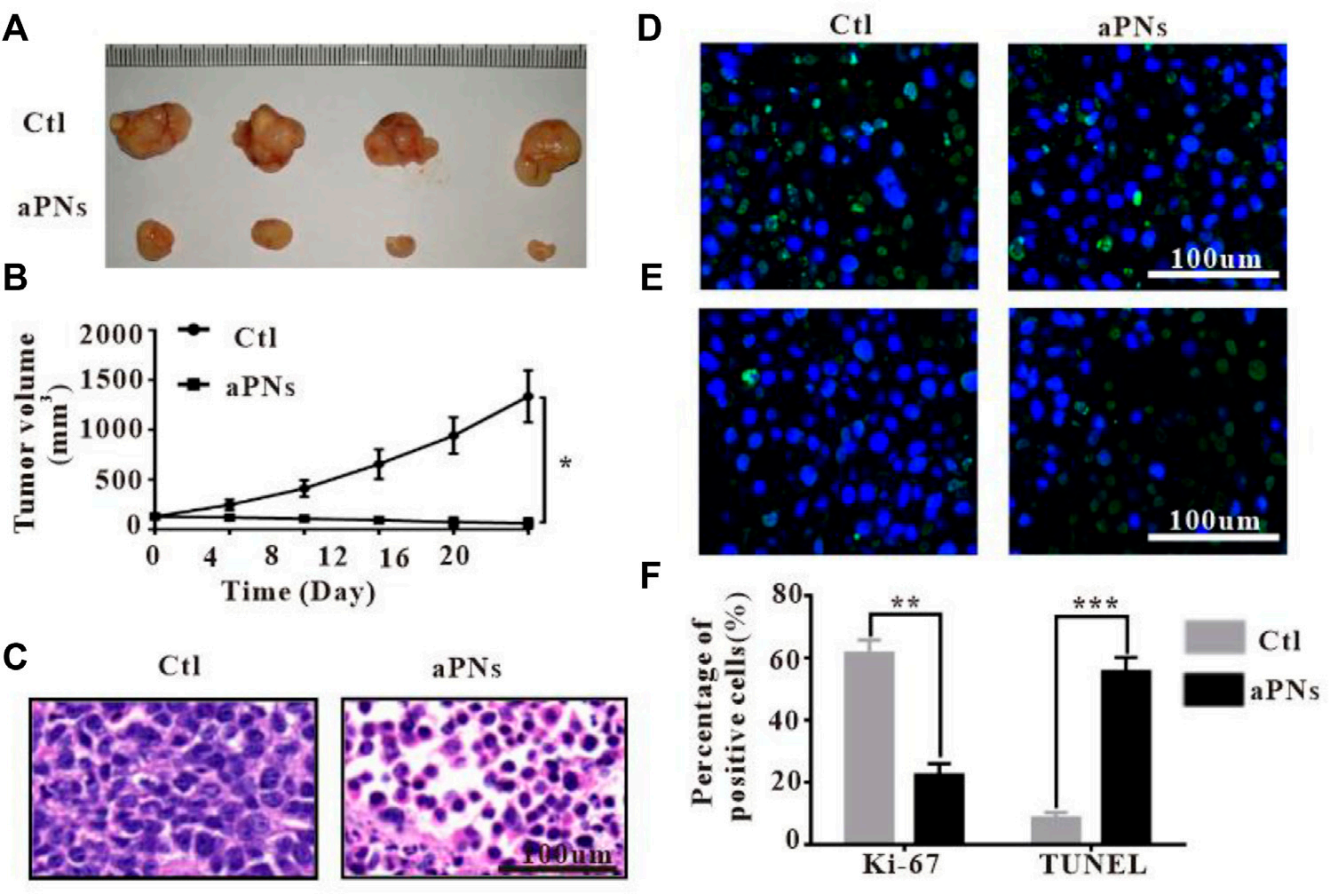
*In vivo* antitumor activity. Ctl: control; aPNs: activated PLTm nanovesicles. **(A)** Representative image of tumor tissues. **(B)** The changes of tumor volume. Data are represented as mean ± SD (*n* = 3), compared with control: ^∗^ *p* < 0.05. **(C)** Histological images of tumor sections, scale bar: 100 μm. **(D)** Histological images of tumor sections after Ki-67 immunofluorescence staining, scale bar: 100 μm. **(E)** Images of tumor tissue after TUNEL immunofluorescence staining, scale bar: 100 μm. **(F)** Statistical analysis of Ki-67-positive and TUNEL-positive cells, respectively, compared with control: ^∗∗^ *p* < 0.01 and ^∗∗∗^ *p* < 0.001.

After treatment with aPNs, the tumor volume dramatically shrank (Figures 3A,B), indicating that aPNs can significantly inhibit tumor growth. The tumor tissue sections stained by HE (Figure 3C) showed that 4T1 cells in the control group grew well, but necrosis of 4T1 cells increased significantly after the treatment of aPNs, indicating that aPNs could significantly inhibit the growth of 4T1 cells.

### 3.4 Ki-67 and TUNEL immunofluorescence in tumor tissue

After treatment with aPNs, Ki-67-positive tumor cells decreased (Figures 3D,F), indicating that aPNs could inhibit the proliferation of tumor cells. TUNEL-positive tumor cells in the aPN group were more than that in the control group (Figures 3E,F), suggesting that aPNs promote the apoptosis of tumor cells.

### 3.5 Immunofluorescence detection of immune cells in tumor tissues

**FIGURE 4:**
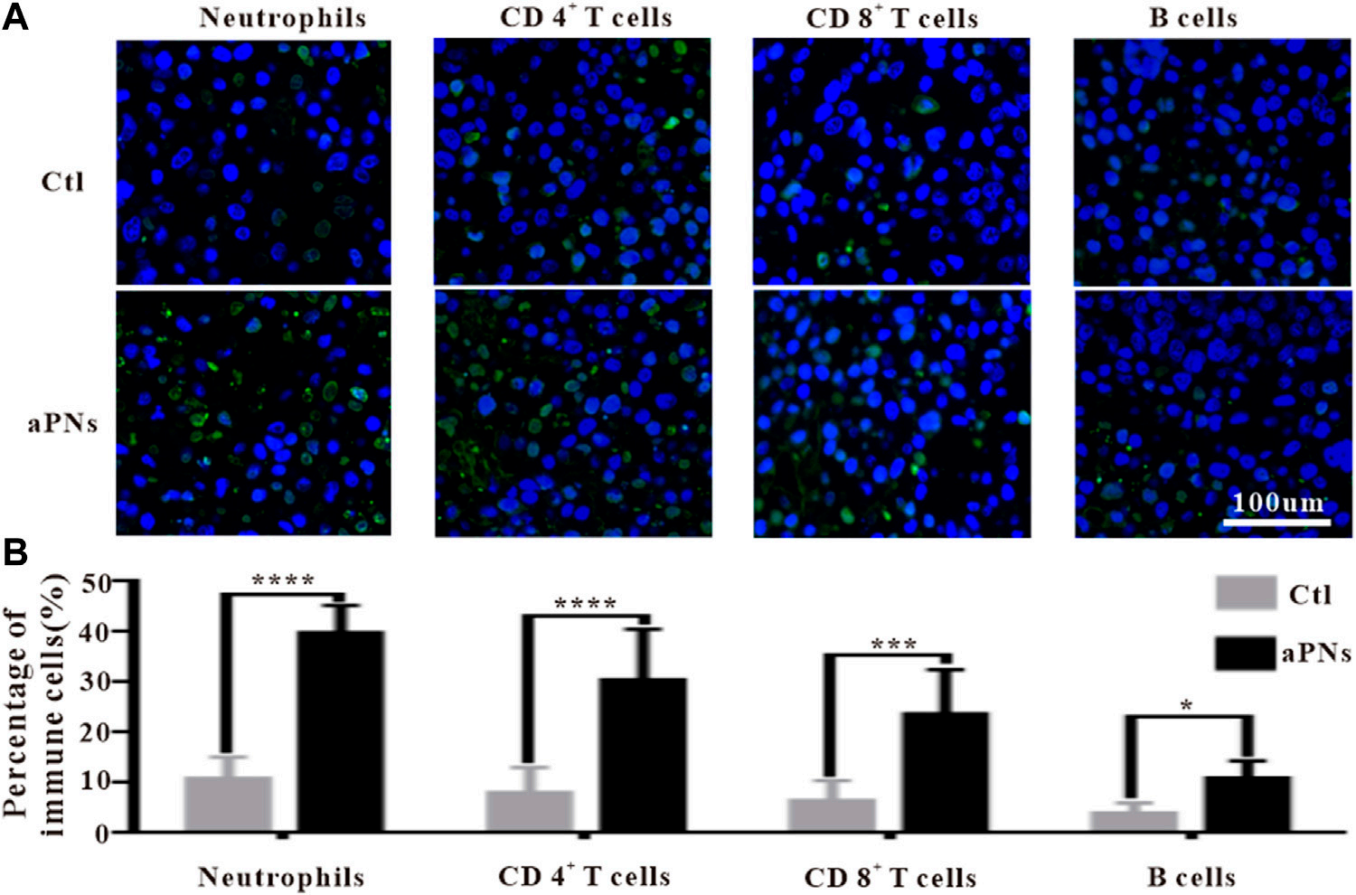
Immunofluorescence images of immune cells. Ctl: control; aPNs: aPLTm nanovesicles. **(A)** Immunofluorescence images of neutrophils, CD4^+^ T cells, CD8^+^ T cells, and B cells, scale bar: 100 μm. **(B)** Statistical analysis of immune cells, compared with control: ^∗^ *p* < 0.05, ^∗∗∗^ *p* < 0.001, and ^∗∗∗∗^ *p* < 0.0001.

Platelets can recruit neutrophils through P-selectin, ICAM-2, and CCL5 (Weber and Springer, 1997; Kuijper et al., 1998; Nagao et al., 2007; von Hundelshausen et al., 2007). Activated PLTm nanovesicles express P-selectin, ICAM-2, and CCL5. Accordingly, we inferred that activated PLTm nanovesicles can also recruit neutrophils. As can be seen from Figures 4A,B, neutrophils (green immunofluorescence) in tumor tissue were dramatically increased after treatment with activated PLTm nanovesicles. It is suggested that tumor-targeted activated PLTm nanovesicles could recruit neutrophils to tumor site.

Neutrophils play an anticancer role by inducing apoptosis of tumor cells, producing antitumor cytokines or cytotoxic reactions. Neutrophils engulfed dead tumor cells and present neoantigens to promote T cells-based adaptive immune response (Huang et al., 2016; Marzagalli et al., 2019). After treatment with aPNs, neutrophils, CD4^+^, and CD8^+^ T cells in the tumor tissues increased significantly (Figures 4A,B), indicating that the presentation of tumor antigen and antitumor immunity were reinforced, thus enhancing antitumor effect.

CD4^+^ T cells play a key role in driving both the antibody response and the cytotoxic CD8^+^ T-cell response by producing IFN-γ, thereby contributing to the formation of an inflammatory environment conducive to antitumor immunity. Moreover, CD4^+^ T cells are important in mediating cytotoxicity. In fact, tumors that express MHC class II molecules, exposed to IFN-γ, may be direct targets of cytotoxic CD4^+^ T cells (Chen and Mellman, 2017). As shown in Figures 4A,B, after treatment with aPNs, CD4^+^ and CD8^+^ T cells obviously increased, and the level of IFN-γ was elevated, indicating enhanced cytotoxicity and antitumor immunity.

**FIGURE 5:**
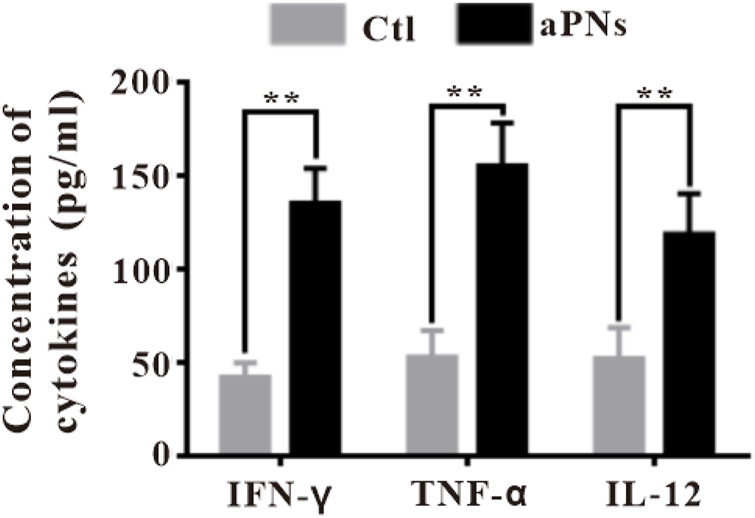
Cytokines ELISA detection in tumor tissues. Ctl: control; aPNs: aPLTm nanovesicles. Data are represented as mean ± SD (*n* = 3), compared with the control group: ^∗∗^ *p* < 0.01.

IFN-γ stimulates the polarization of neutrophils into anticancer phenotype characterized by cytotoxicity against tumor cells and the acquisition of antigen-presenting cell characteristics (Eruslanov et al., 2014; Singhal et al., 2016), and promotes antitumor adaptive immunity (Governa et al., 2017). Neutrophils and IFN-γ in the tumor tissue were significantly increased after aPN treatment (Figures 4A, 5), indicating enhanced antitumor immunity and cytotoxic activity.

B cells are positively correlated with the reduction of tumor volume (Cunha et al., 2012). Compared with cases with visceral metastases, there were more B cells in inflammatory infiltrations of non-metastatic melanoma or tumors that only invaded lymph nodes, indicating that higher B-cell count was correlated with better prognosis (Ladanyi et al., 2011). Figure 4A showed that B cells in the tumor tissue were significantly increased after aPN treatment, compared with the control group, indicating enhanced antitumor effect and a better prognosis.

CD4^+^ T helper cells promote B-cell-mediated immune response (Mitsdoerffer et al., 2010), facilitate the migration and activation of neutrophils (Pelletier et al., 2010), and control the activation and proliferation of CD8^+^ T cells (Martin-Orozco et al., 2009; Ankathatti Munegowda et al., 2011). IFN-*γ* and TNF-α produced by CD4^+^ T cells (Kryczek et al., 2009) play direct cytotoxic role on cancer cells, which are related to the activation of innate and adaptive immunocyte, thus motivating antitumor immune response (Alizadeh et al., 2013). Tumor tissue infiltration of neutrophils, CD8^+^ T, CD4^+^ T, and B cells significantly increased after the treatment of aPNs (Figures 4A,B), suggesting enhanced antitumor immune activity. In addition, as shown in Figure 5, TNF-*α* and IFN-*γ* levels increase, indicating increased anticancer activity.

Activated CD8^+^ cytotoxic T lymphocytes (CTLs) induce apoptosis of tumor cells directly by secreting granzyme B and perforin; or act on tumor cells indirectly by releasing cytokines, such as IFN-γ and TNF, resulting in consecutive appearance of antigens and the proliferation of T cells (thor Straten et al., 1999; Durgeau et al., 2018; Marzagalli et al., 2019). Compared with the control group, CD8^+^ CTL in tumor tissues increased significantly in the aPN group (Figure 4), suggesting enhanced antitumor effect.

### 3.6 Cytokine ELISA detection of tumor tissues

IFN-γ, produced by adaptive and innate immunocytes, including T cells and NK cells, serves as a potent immunomodulatory cytokine (Gattoni et al., 2006). IFN-γ modulates a variety of effects, including antiproliferative, anticancer, and adaptive immunity, and has been reported to induce apoptosis and inhibit cell proliferation (Stark et al., 1998; Gattoni et al., 2006). In the aPN group, the IFN-γ level elevated (Figure 5), Ki-67-positive cancer cells decreased (Figures 3D,F), and TUNEL-positive cancer cells increased (Figures 3E,F), indicating enhanced antitumor immunity and anticancer effect.

TNF-α secreted by tumor cells promotes cell apoptosis and inhibits survival in cancer (Szlosarek et al., 2006; Balkwill, 2009) and controls cancer (Shankaran et al., 2001; Willimsky et al., 2008). These identified effects agreed with our finding that the level of TNF-α in tumor was significantly increased after treatment with aPNs (Figure 5), accompanied by a significant decrease in tumor volume.

IL-12, secreted from various immunocytes, such as neutrophils and B cells, stimulates the production of IFN-γ by T cells (Conlon et al., 2019), playing a remarkable antitumor effect (Engel and Neurath, 2010). IL-12 stimulates the activity of cytotoxic T cells and promotes B-cell survival (Conlon et al., 2019), acts as a central coordinator of immune responses, and bridges innate to adaptive immunity in humans (Langrish et al., 2004). Moreover, IL-12 mainly acts on T cells, promoting cytotoxic activity of CTLs and inducing the production of cytokines, including IFN-γ and TNF-α (Gao et al., 2019). The number of B cells increased significantly after aPN treatment (Figure 4), suggesting B-cell-mediated antitumor immune enhancement. In addition, the levels of IL-12, TNF-α, and IFN-γ increased in tumor tissues in the aPN group (Figure 5), indicating enhanced antitumor immunity and anticancer effect.

## 4 Conclusion

**Scheme 1:**
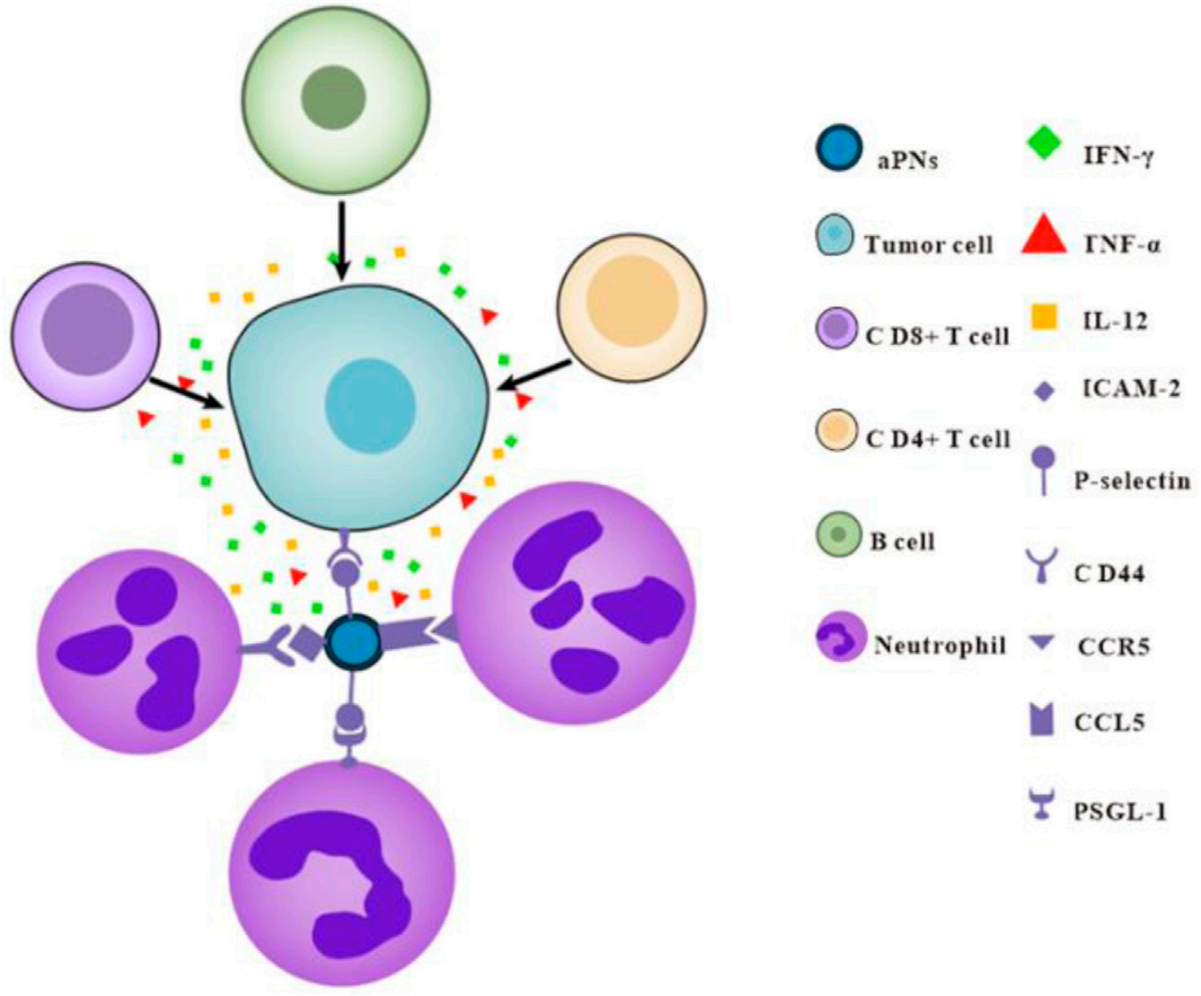
Schematic diagram of aPNs enhancing antitumor immunity.

Activated platelet membrane nanovesicles, which retain almost all of the platelet-proteins, target tumor tissues through P-selectin and CD44, and mediate neutrophil recruitment through P-selectin, ICAM-2, and CCL5. Subsequently, neutrophils increase the number of CD8^+^, CD4^+^T cells, and B cells in the tumor and promote the release of IFN-γ, TNF-α, and IL-12; thus, the antitumor immunity and anticancer effect was enhanced (Scheme 1). Based on our study, platelet membrane nanovesicles are expected to provide a new tool for tumor treatment and open up new ideas for cancer therapy.

## Data Availability

The original contributions presented in the study are included in the article/supplementary material; further inquiries can be directed to the corresponding author.
